# Policy measures to expand home visiting programs in the postpartum period

**DOI:** 10.3389/fgwh.2022.1029226

**Published:** 2023-01-04

**Authors:** Binh Phung

**Affiliations:** ^1^ Department of Pediatrics, Oklahoma State University Center for Health Sciences, Tulsa, OK, United States; ^2^ Department of Epidemiology and Public Health, Yale University, New Haven, CT, United States

**Keywords:** home visiting programs, policy measure, maternal mortality, black maternal health, post-roe era, pregnancy-related deaths, postpartum care, postpartum period

## Abstract

The postpartum period is characterized by a myriad of changes—emotional, physical, and spiritual; whilst the psychosocial health of new parents is also at risk. More alarmingly, the majority of pregnancy-related deaths in the U.S. occur during this critical period. The higher maternal mortality rate is further stratified by dramatic racial and ethnic variations: Black, brown, and American Indian/Alaska Native indigenous people have 3–4x higher rates of pregnancy-related deaths and severe morbidity than their White, non-Hispanic, and Asian/Pacific Islander counterparts. This policy brief explores how expanding evidence based home visiting programs (HVPs) and strengthening reimbursement policies that invest in such programs can be pivoted to optimize the scope of care in the postpartum period.

## Introduction

The postpartum period is defined by the World Health Organization (WHO) as the period beginning immediately after delivery of the baby and extending up to 6 weeks (1). This critical time is characterized by a range of physical, lifestyle, and emotional changes, resulting in increased needs for support for birthing people and their families (2). Cultures worldwide have traditionally prescribed a 30–40-day (postpartum) period of rest and recovery, with the birthing individual being surrounded by their family and/or community members (3). While these traditions and practices have been largely sustained elsewhere in the world, this timeframe punctuates a period devoid of both formal medical and informal social support for many people living in the United States (U.S.), especially low-income black and brown birthing individuals. Furthermore, postpartum care in the U.S. is often fragmented and communication is inconsistent from the inpatient to outpatient settings (4). Most birthing people ultimately end up navigating the postpartum transition on their own. This lack of attention to health needs and support is of concern given that more than half of pregnancy-related deaths occur in the postpartum period (5).

Disparities are not unique to the U.S., however. In the United Kingdom, which has universal health coverage, maternal deaths were five times more common among Black women (in the pre-COVID period) and two times more common among Asian women (6). Achieving equity in maternal outcomes remains a global challenge. As the U.S. continues to grapple with newly imposed limitations on reproductive health rights after the Supreme Court's consequential ruling on Dobbs v. Jackson Women's Health Organization,^1^ this is an opportune moment to address health disparities that may soon be amplified by the loss of human reproductive rights. What existing infrastructures should be reconceptualized and/or new legislations be implemented to curtail the rising *maternal*^2^ mortality rates and provide ongoing support for pregnant/birthing people? This policy brief explores how expanding evidence-based home visiting programs (HVPs) and strengthening reimbursement policies that invest in such programs can be pivoted to optimize the scope of care in the postpartum period.

## Postpartum and pregnancy-related deaths

The terms postpartum and postnatal (period) are often used interchangeably in literature but in this manuscript, “postpartum” refers to issues pertaining to the birthing individual and “postnatal” refers to those concerning the baby (8). The postpartum period is characterized by a myriad of changes—emotional, physical, and spiritual (9); whilst the psychosocial health of new parents is also at risk (10). Postpartum care during this period recognizes that both the baby and their parent(s) may need an additional 3 months to adapt outside the womb and to fully recover from the pregnancy/birthing process, respectively. The postpartum period has recently been acknowledged as the “4th trimester” by the American College of Obstetricians and Gynecologists (11).

Pregnancy-related mortality, as defined by the WHO, refers to the “death of an individual while pregnant or within 42 days of termination of pregnancy, irrespective of duration and site of pregnancy, from any cause related to (or aggravated by) the pregnancy or its management, but not from accidental or incidental causes (12). Maternal mortality statistics in the U.S. are commonly reported by three measures (e.g., pregnancy-associated death, pregnancy-related death, and maternal mortality). Refer to Appendix 1A for descriptions. The specific measure known as “pregnancy-related deaths”, which is only reported by the CDC (as a ratio of deaths per 100,000 births), can broadly account for all deaths occurring during pregnancy, delivery, and up to 1 year post birth (10).

The majority of pregnancy-related deaths in the U.S. occur in the postpartum period. Trost et al. corroborated on this alarming trend during 2017–2019: 22% of maternal deaths occurred intrapartum, 25% on the day of delivery, 23% on days 7–42 postpartum, and 30% 43–365 days postpartum (13). Thus, more than half (53%) of pregnancy-related deaths (for which timing in relation to pregnancy is known) occurred 7–365 days postpartum (13). From another perspective, consider that for every 100,000 live births, 20 people died while pregnant or within 42 days of the end of their pregnancy in 2019 (14, 15). Among the pregnancy-related deaths determined by data collected from Maternal Mortality Review Committees in 36 different states, the overall percentage (of preventable deaths) was an astounding 84% (13).

## The impact of HVPs in the U.S.

Home visiting programs (HVPs) are one of several service strategies, which was bolstered in funding by the Affordable Care Act 2010, and rooted in a comprehensive, early childhood system that promotes caregiver, infant, and early childhood health, safety, and development (14). Depending on the type of program, the “home visitor” can a registered nurse, a midwife, a social worker, or a child developmental specialist. HVPs often serve high risk families and address health disparities by coordinating care delivery, initiating important referrals, or assessing for other adjacent needs during the postpartum period (14). When HVPs utilize culturally responsive and community-driven approaches to support at risk, low-income, and underserved families, they can be strategically positioned to improve the overall coordination of care and address racial/ethnic disparities (15). Home visits by a nurse or a midwife are associated with improved mental health (16), breastfeeding challenges (17), and reduced health care costs (15).

Since 2009, the Department of Health and Human Services (DHHS) has conducted a continuing assessment review program called the Home Visiting Evidence of Effectiveness (HomVEE), which aimed to publish transparent reviews of all HVPs across the country. HomVEE has shown that early childhood evidence based HVPs can: (1) improve overall caregiver and infant health outcomes, (2) increase postpartum depression screenings, (3) reduce child abuse and neglect, (4) promote child development and school readiness, (5) support positive parenting methods, and (6) improve coordination/referrals for appropriate community resources (15).

For the fiscal year 2020, the National Home Visiting Resource Center (NHVRC) estimated that more than 17.6 million pregnant/birthing people and their families could have benefited from services provided by HVPs (18). These individuals and their families met the following inclusion criteria (i.e., *stressors*) as defined by the NHVRC: (1) raising an infant <1 year old, (2) having low income (below federal poverty threshold), (3) being a single parent, (4) being pregnant and <21 years old, and (5) not having a high school diploma. Of the 17.6 million people, just under 298,000 individuals were actually served by HVPs (19). Among these families, 25% were Black and 29% were Hispanic or Latino. Expanding the capacity of HVPs to reach more families (covered by Medicaid), especially those with multiple *stressors*, could be one solution to rectify the ongoing structural, social, and geographic factors that exacerbate health disparities (by way of race, ethnicity, socioeconomic position, immigration status).

## Increasing engagement and strengthening postpartum care

Postpartum care is intended to ensure the physical and emotional recovery of birthing individuals and their babies (20). Hence, postpartum home visits are currently covered by national insurance plans and guaranteed within 1 week of delivery in 10 developed countries (e.g., Australia, Canada, France, Germany, Netherlands, New Zealand, Norway, Sweden, Switzerland, United Kingdom) (21, 22). In addressing the pregnancy-related mortality crisis, the American College of Obstetricians and Gynecologists has advocated that “postpartum care should be an ongoing process […] with routine post-partum visits starting as early as the first 3 weeks” after delivery (7). In the current postpartum care delivery model, pregnant/birthing people are routinely discharged from the hospital within 24–48 h after giving birth. Their first postpartum visit is usually just a one-time follow-up encounter scheduled about 6 weeks post-delivery. Earlier access to care (e.g., home visitation) varies significantly across the U.S., with some states providing visits only for its Medicaid beneficiaries.

As many as 40% of birthing people do not attend a postpartum visit in the U.S. (23). Attendance rates are lower among populations with limited resources, which further contributes to health disparities (24). Black and indigenous American Indian/Alaska Native (AI/AN) people have 3–4x higher rates of pregnancy-related deaths and severe morbidity than White, non-Hispanic, and Asian/Pacific Islander people (22). The top six most frequent underlying causes of pregnancy-related deaths include mental health conditions, excessive bleeding (hemorrhage), cardiac/coronary conditions, infection, thrombotic embolism, and cardiomyopathy (13). Cardiomyopathy, thrombotic pulmonary embolism, and hypertensive disorders of pregnancy contributed more to pregnancy-related deaths among black women […] while hemorrhage and hypertensive disorders of pregnancy contributed more to pregnancy-related deaths among AI/AN women than white women (25). Early identification and management of hypertension is pivotal because these birthing people are at increased risk of stroke, seizures, pulmonary edema, renal failure, congestive heart failure, and death (26).

Various aspects of the home visiting model can be incorporated in the postpartum period such as blood pressure monitoring (27), text-messaging support (28), and app-based support (29). For instance, blood pressure monitoring is strongly recommended for individuals with hypertensive disorders of pregnancy no later than 7–10 days after delivery (30); and those with severe hypertension should be seen with 3 days after delivery (12). Increasing engagement and subsequent postpartum visits/attendance could facilitate early identification of and treatment for the common causes of pregnancy-related deaths mentioned above. Early assessments could help to strengthen postpartum care for cardiac/coronary conditions since more than half of postpartum strokes occur within 10 days of delivery (31). In addition, home visits may be beneficial people with other high risk of complications, (e.g., postpartum depression, cesarean or perineal wound infection, lactation problems), and/or other chronic medical conditions (e.g., seizure disorder).

## Proposed policy measures

On April 13, 2021, President Joseph Biden issued the first-ever proclamation for “Black Maternal Health Week”, which emphasized the importance of recognizing and addressing disparities in maternal health. The White House (WH) administration then released a detailed outline which comprised of (1) funding requests made to Congress (related to health care delivery for pregnant people), (2) recommendations for individual states to expand Medicaid coverage for pregnancy/postpartum care, and (3) new funding grants for rural obstetrics care (32). On December 7, 2021, during the inaugural “Maternal Health Day of Action”, Vice-President Kamala Harris announced additional commitments from the WH administration to further support safe pregnancies/childbirth and reduce mortality in the postpartum period (32).

Building on this momentum and increased awareness of maternal health, the following policy brief will present **5 policy measures** that can be implemented to help states expand their capacity and funding for HVPs. Optimizing care and support for postpartum families will require funding and policy changes. Thus, four of the five policy measures are strictly meant to facilitate reimbursement to support postpartum care as an ongoing process, rather than an isolated office encounter.

(1) streamline waiver application process & enhance Medicaid coverage for HVPs
(2) leverage 10% increase in Federal Medical Assistance Percentage (FMAP)
(3) clarify federal braided funding options for HVPs
(4) increase Maternal, Infant, and Early Childhood Home Visiting (MIECHV) funding for tribal populations from 3% to 10%
(5) tech innovations to create “digital care packages” to assist birthing people navigate their postpartum care

### Policy measure #1

Streamline federal waiver application process and enhance Medicaid eligible benefits coverage for HVPs.

○ **Context**: Medicaid is an important lever to pivot and expand funding options for HVPs. According to the Health Resources and Services Admnistration’s Joint Information Bulletin, HVPs are not formally specified as *covered benefits* under Medicaid (33). States may, however, choose to apply for various Medicaid funding options to help cover for portions of HVPs. For example, the Joint Information Bulletin identified 3 core foundational elements of HVPs that could be considered “eligible services” for enrolled Medicaid participants: (1) screening, (2) case management, and (3) family support, counseling, and skills training (34). States may apply for federal waivers and/or demonstration projects for additional funding resources. Waivers will allow individual states to adapt their Medicaid funding agreements, which may vary among different federal Medicaid-state requirements (35). The main caveat is that most federal waivers are time consuming with lengthy application submission/approval processes.
○ **Pros**: Most pregnant individuals and their children served by HVPs are already enrolled participants in Medicaid and CHIP (Children’s Health Insurance Program)—which is a key advantage point to explore how Medicaid can be leveraged through legislative processes (Refer to Appendix 1B—*Government funding infrastructure & legislative/administrative processes* for more background on funding.)
○ **Cons**: Medicaid funding in certain states (i.e., “non-Medicaid expansion states”) is limited and directing federal funding toward ancillary services like HVPs can be politically challenging for state legislators and stakeholders. This notion of enhancing Medicaid criteria for eligible services such as HVPs would be far less feasible for states that have not participated in Medicaid expansion.
○ **Limitations**: The major funding source for HVPs in all 50 states is currently provided by Maternal, Infant, and Early Childhood Home Visiting (MIECHV) (36) (Refer to Appendix 1B for more details).
○ **Tradeoffs**: Assuming that Congress will re-authorize MIECHV funding for 5 more years, there are some specific steps that can be taken to accomplish key policy measures #1:
  (1) Provide clearer instructions, simpler online waiver templates for home visiting state agencies on how to apply for federal waivers for operational funding of HVPs (that’s not covered by MIECHV).
  (2) Streamline the Medicaid selection criteria process for states that elect to apply “eligible services” covered under Medicaid in order to expand the capacity and access of HVPs.
  (3) The Centers for Medicare & Medicaid Services (CMS) could provide instructions for state agencies on how to effectively utilize the Delivery System Reform Incentive Payment program, which is a newer type of supplemental payment system that CMS can directly dispense reimbursements for “eligible services” rendered on behalf of Medicaid-enrolled pregnant individuals and their children.
  (4) Finally, ensure that Medicaid reimbursement rates will also cover for those initial administrative and professional startup costs of HVPs (such as training of home visitors, data management, supervision, and other activities).

### Policy measure #2

Leverage a 10% increase in the Federal Medical Assistance Percentage (FMAP) for Medicaid expenditures on HVPs for a period of 5 years.

○ **Context**: The COVID-19 pandemic has shown us that Medicaid can be leveraged to fill the coverage gaps for postpartum individuals. One such example was a provision created in the Families First Coronavirus Response Act. This provision included a *continuous coverage* option that linked receipt of enhanced federal Medicaid and CHIP funding to a prohibition on involuntary disenrollment from Medicaid (37). As a consequence of this *safety net* provision, which was crafted largely due to the economic uncertainties caused by the COVID-19 pandemic, Medicaid and CHIP enrollment has now grown to a historic high of 89 million (38) according to data published for the month of May 2022. This provision was an influential forward-thinking concept and considered to be a step in the right direction to disrupt macro-level policies. Furthermore, the objective of this provision intended to unravel endemic vulnerabilities and disparities that have disproportionately impacted Black women and birthing people of color.
○ **Pros**: CMS recently announced a Maternity Care Action Plan (July 2022)—a proposal for more holistic and coordinated approach to maternal health care (39). This is congruent with the current White House administration’s track record of backing similar policies aimed at improving maternal health.
○ **Cons**: In general, individual states’ Medicaid programs face quarterly/annually budgetary funding cuts and restrictive legislative actions. Because most Medicaid enrollees are women (and children) of color, any broad policy changes to the federal funding streams could potentially undermine downstream payment structures, disproportionately impact marginalized populations, and perpetuate structural racism (socioeconomic/healthcare).
○ **Tradeoffs**: In the immediate aftermath of the Dobbs ruling, we know that access and regulations to reproductive health and related services has now been reapportioned to the jurisdictions of states and local governments. The long-term consequences of the Dobbs ruling will undoubtedly have implications for future state fiscal funding budgets. However, leveraging a policy that will guarantee an increase of 10% in FMAP for Medicaid expenditures on HVPs (and/or similar outreach services) could temporarily motivate states to scale up efforts to expand HVPs sooner.

### Policy measure #3

Clarify how federal funds can (or cannot) be braided for HVPs.

○ **Context**: The lack of parity in expanded Medicaid coverage for HVPs across different states has potential to shape the social determinants of health.
○ **Pros**: States could benefit from better clarification on how federal funds can (or cannot) be braided or supplemented. The White House administration should provide clearer written guidance for states—making for simpler administrative separation of (and accounting for) such federal funds.
○ Diversification of federal funding sources can correspondingly increase home visiting capacity, support quality, and optimize the use of HVPs for prevention/intervention purposes.

### Policy measure #4

Increase MIECHV funds for tribal populations from 3% to 6%.

○ **Context**: Redressing structural racism and health inequities in maternal outcomes, in part through HVPs, could become a key driver for government-funded health insurance programs because Medicaid currently covers, on average, 6 in 10 births from Black, Latino, and American Indian/ Alaskan Native (AI/AN) people (33).
○ **Pros**: Tribal MIECHV programs have provided home visiting services for 23 grantees (including Indian tribes, consortia of tribes, tribal organizations, and urban Native American organizations). The tribal home visiting program is funded by a 3% set-aside from the larger MIECHV program (40). This policy measure does not need a benefit/cost analysis or tradeoffs because tribal MIECHV programs are critical life-saving outreach services for many indigenous AI/AN birthing people and their families.

### Policy measure #5

Encourage tech innovations to create “digital care packages” to assist birthing people navigate their postpartum care.

○ **Context**: Modern advancement in *geospatial technology* (i.e., public access to large population clinical data) can provide contextual information and supplement our understanding of the underlying drivers of maternal health disparities (41). Health information technology tools such as electronic health records, patient portals, telemedicine, and machine learning algorithms can be used to alert birthing people about the cultural, ethnic, and geographic disparities of the surrounding communities in which they live. The White House administration can provide incentives and/or grant funding for tech companies to build privacy compliant social networking interfaces or virtual patient advocates/avatars within existing electronic health systems, patient portals, and/or telemedicine models. De-identified personal clinical data can be collected and aggregated according to the specific demographics of birthing individuals (i.e., neighborhoods, communities, built environment, communicable diseases, and common health risks). Community-level geocoded data can then be translated into personalized *digital care packages* (e.g., tailored lists of local/state HVPs, clinicians, and community resources) to help pregnant/birthing people living in those specific communities navigate their postpartum care.
○ **Pros**: Collection of demographic data, prediction and mitigation of postpartum health risks from groups of birthing individuals living in similar communities are innovative tech solutions. One digital platform that has previously been built using the pilot web tool called the “Maternal Data Center” now has up to 300 participating hospitals across Washington, Oregon, and California. This webtool generates data and publishes performance metrics on maternity care services in real time.
○ **Cons**: Tech innovations designed to optimize postpartum care delivery assume that all participants have access to the internet, possess similar health literacy, self-efficacy, and social support. These assumptions ignore the intersectionality of cultural conditions (such as race, gender, health literacy, and social networks) and might fall into the culturalism trap (e.g., those who can afford internet access versus those who cannot).
○ **Limitations**: The time, effort, resources required to develop sharable, protected health information, and disseminate community-level geocoded digital care packages could be costly and labor intensive. Limited availability of tech support and lack of systems interoperability among different electronic health systems make this policy measure less feasible.^2^
○ **Tradeoffs**: Instead of tech companies, the government can perhaps provide direct financial incentives (e.g., grants, awards, etc.) for state agencies to innovate their existing home visiting programs, formulate technical solutions, and promote cross-agency collaboration to securely share digital care packages with community partner organizations.

## Conclusion

While the causes driving maternal mortality rates in the U.S. are multifactorial, strengthening reimbursement policies that invest in HVPs is a necessary prerequisite step to optimize postpartum care for all birthing people. The WHO has recommended at least 4 health contacts in the first 6 weeks post-delivery (20), and in many Northern Western European nations, birthing people (and their infants) receive a home visit shortly after delivery, or they can rest and receive support in maternity homes (42). In contrast, in the U.S., HVPs are not routinely incorporated into the postpartum care delivery model. Birthing people typically have a single office-based physician follow-up visit, and some don't have one at all (11). As one population health study has noted, the lack of policies substantially benefitting early life in the U.S. constitutes a grave social injustice: those who are already most disadvantaged in our society bear the greatest burden (43). During Roe, the U.S. was dealing with the syndemic of COVID-19 and unequal macro-level policies (i.e., policies impeded by social determinants, cultural conditions, and structural/systemic racism) that likely exacerbated underlying inequalities in maternal outcomes. In the wake of the Dobbs ruling, the health equity crisis affecting low-income birthing people of color is once again highlighted in America. Evidence-based HVPs is just one solution, at least for the short-term period, that can help to address the health disparities through comprehensive services like coordinating care, initiating referrals, providing maternal education/familial support, and implementing important health, physical, mental assessments during the postpartum period. The first 4 key policy measures represented short-term legislative solutions that leveraged governmental infrastructure and federal funding streams. Policy measure #5 adopted a longer-term (outside the box) approach to encourage tech innovation in the maternal health space. These policy measures are timely and relevant legislative actions that the federal government, state/local policymakers, and connected stakeholders can consider immediately. This policy brief contributes to the larger discussion on reproductive rights and elevates the urgent call for equitable access to postpartum health care for everyone. Multi-pronged strategies (i.e., legal reforms, policy actions) and longer-term solutions (e.g., ways to simultaneously link birthing people, their infant, and family to a variety of additional services) must always be centered around the lived experiences and supported by the voices of those most affected by disparities—low-income Black, indigenous AI/AN, and brown individuals. Their voices cannot afford to wait much longer for legislators to dutifully discuss the pros, cons, and tradeoffs regarding policies/regulations that have already impacted their lives.

## Footnotes

^1^The recent U.S. Supreme Court's ruling on Dobbs v. Jackson Women's Health Organization was issued on June 22, 2022 [7], and instantaneously reinscribed denial of human rights and equitable access to reproductive health care for all child-bearing individuals in America. The broader impact of the Dobbs decision is beyond the scope of discussion for this brief, we won't understand its cataclysmic consequences for years to come. However, we can agree, assuredly, that overturning Roe v. Wade will restrict access to reproductive health care for low-income individuals of child-bearing capacity (and their families).

^2^The author acknowledges that language regarding gender is in flux (e.g., terminology such as maternal health, women's health, pregnancy-related mortality, and maternal mortality). Pregnancy and childbirth occur among people who are biologically female whose gender identity may or may not be that of a woman. Terms such as “birthing person”, “birthing individuals”, or “birthing people” are more inclusive than “pregnant women”, yet most maternal health data in the U.S. and across the world continue to be collected and reported with traditional terms. This policy brief uses a combination of gendered and gender-neutral terms based on the available data used for reporting.

## Appendix

### Appendix 1A: Maternal mortality statistics

Maternal mortality statistics are reported by 3 main data collection systems in the United States: (1) National Vital Statistics System (44), (2) Pregnancy Mortality Surveillance System (reported by the Centers for Disease Control & Prevention as the number of pregnancy-related deaths per 100,000 live births), and (3) individual states’ Maternal Mortality Review Committees (34) which involve multiple stakeholders.

### Appendix 1B: Government funding infrastructure & legislative/administrative processes.

Under the Affordable Care Act, the federal government established the MIECHV program, which represents the largest source of federal funding for HVPs (45). Some states have utilized funding from MIECHV to identify high-risk target populations and expand evidence based HVPs where applicable. While the MIECHV program is a critical federal investment, additional funds are needed to reach all the families who could benefit from these services (15). The processes would include proposals from the executive branch (White House) made to the legislative branch (Congress). If approved, then U.S. agencies like CMS and Department of Health and Human Services (DHHS) could implement these policy measures. States would also need to submit a formal Medicaid budget/plan proposal to CMS to help fund HVPs or submit a waiver application. States must identify sources of funding for their state share of Medicaid matching funds. Finally, states would need to implement outreach programs and provide appropriate training of staff for HVPs.

Fiscal relief funds from the American Rescue Plan have been deployed to improve the lives of infants and young children during the COVID-19 pandemic. Specifically, for HVPs, the ARP provided $150 million (in additional to the funding from MIECHV). There was also $350 million from DHHS appropriated to states—to strengthen maternal and child health by expanding home visiting services to families in need, addressing health disparities in infant mortality, and strengthening data reporting on maternal mortality (15).

### Appendix 1C: Main stakeholders: Opposers vs. supporters of expanding HVPs

State policymakers and non-Medicaid expansion states were identified as potential **opposers** of the expanding HVPs. A recent 2019 Mother and Infant Home Visiting Program independent evaluation (conducted by the Office of Planning, Research, and Evaluation of the Administration for Children and Families) examined the outcomes of various HVPs. Opposers of Medicaid expansion might point to this evaluation outcome study because it found no statistically significant differences in health and well-being outcome measures when stratified across race and ethnicity even though HVPs were implemented with fidelity (37). This was not a surprise because the HVPs that were selected for evaluation were not intentionally designed to mitigate racial and ethnic disparities. Other home visiting models have successfully achieved specific outcomes based on the intended goals/intentions they were designed to accomplish. For example, “Parents as Teachers”^3^ had the largest increase in parental supportiveness (46) and “Nurse-Family Partnership” programs had the largest reductions in emergency department visits for children (47). Moving forward, expanding quality HVPs with clear anti-racist objectives and culturally competent practices is essential in order to reach more mothers, infants, and children of color (38).

Pregnant/birthing low-income people, the White House administration, and the Department of Human & Health Services (DHHS) were identified as potential **supporters** of expanding HVPs. Aside from addressing the social and economic determinants that coalesced to reproductive health inequalities and injustices, HVPs has been shown to yield *net cost savings* when aligned with value-based payment structures that can sustain a successful model of prenatal care (48). For example, a North Carolina study showed that when pregnant individuals (who were covered by Medicaid for least part of their pregnancy) were enrolled in the state's Baby Love Maternity Care Coordination Program—an HVP that coordinated prenatal visits, mental health counseling, and childbirth education—the state experienced a significant decline in preterm birth risk compared to the control group (49). The Nurse-Family Partnership model has shown yields of $1.25–$5.70 for every $1 invested, with *net cost-saving* benefits to that state of between $10,000 and $41,000 per child served in the U.S. (50).
